# A Large-Scale Analysis of Impact Factor Biased Journal Self-Citations

**DOI:** 10.1371/journal.pone.0161021

**Published:** 2016-08-25

**Authors:** Caspar Chorus, Ludo Waltman

**Affiliations:** 1 Department of Engineering Systems and Services, Delft University of Technology, Delft, The Netherlands; 2 Centre for Science and Technology Studies, Leiden University, Leiden, The Netherlands; Katholieke Universiteit Leuven, BELGIUM

## Abstract

Based on three decades of citation data from across scientific fields of science, we study trends in impact factor biased self-citations of scholarly journals, using a purpose-built and easy to use citation based measure. Our measure is given by the ratio between i) the relative share of journal self-citations to papers published in the last two years, and ii) the relative share of journal self-citations to papers published in preceding years. A ratio higher than one suggests that a journal’s impact factor is disproportionally affected (inflated) by self-citations. Using recently reported survey data, we show that there is a relation between high values of our proposed measure and coercive journal self-citation malpractices. We use our measure to perform a large-scale analysis of impact factor biased journal self-citations. Our main empirical result is, that the share of journals for which our measure has a (very) high value has remained stable between the 1980s and the early 2000s, but has since risen strongly in all fields of science. This time span corresponds well with the growing obsession with the impact factor as a journal evaluation measure over the last decade. Taken together, this suggests a trend of increasingly pervasive journal self-citation malpractices, with all due unwanted consequences such as inflated perceived importance of journals and biased journal rankings.

## Introduction

It is well known that the impact factor–arguably the single most important measure for assessing the quality or impact of scholarly journals–is vulnerable to all sorts of manipulation ([1], [2], [3]). The impact factor can for instance be manipulated by publishing large numbers of so-called non-citable articles ([4], [5], [6]). Manipulation is also possible by publishing editorials with many journal self-citations to recently published papers ([7], [8]) or by participating in citation cartels ([9], [10], [11]). Another source of manipulation which received considerable attention lately, is that of coercive journal self-citation ([12], [13], [14], [15]). The phenomenon of coercive journal self-citation has been discussed in various fields of science, ranging from Medicine ([13]) to Information Systems ([16]), Social Sciences ([14]), Sociology ([17]), and Transportation ([18]). Several forms of coercive journal self-citation can be distinguished: for example, a journal may highlight on its website that it requires authors to position their paper with respect to recent papers published in that same journal. Or a journal’s editor, who may perhaps feel pressured by his or her publisher to increase the impact factor of his or her journal, may request authors of conditionally accepted papers to add references to papers recently published in the journal. In response to (anticipated) coercive citation practices, authors may behave strategically by adding references to papers recently published in the journal to which they plan to submit their work, to increase the chance of surviving the (editorial) review process.

This paper studies the phenomenon of ‘impact factor biased journal self-citations’. We use this term to refer to the phenomenon of journals having a disproportionally large proportion of journal self-citations to the past two years, which are the years that determine the impact factor of a journal, relative to their proportion of journal self-citations to earlier years. Impact factor biased journal self-citations may result from coercive citation practices and authors’ strategic response to such (anticipated) practices. They may also result from journal self-citations in manipulated editorials or simply from a tendency of editors to be more inclined to accept submissions that include many references to papers published recently in their journal. However, as we will discuss, there are also legitimate mechanisms that may result in impact factor biased journal self-citations.

Our study contributes to the growing literature on impact factor manipulation in the following two ways. First, we present an easy to use measure of impact factor biased journal self-citations. As we will show, by means of an empirical comparison of our measure with recently reported results of an author survey into coercive journal self-citation malpractices, the measure provides a useful tool to identify (from a large set of candidate journals) a small subset of journals that are relatively likely to engage in self-citation malpractices. As such, it provides a practical tool for a first diagnosis of, for example, the coercive journal self-citation phenomenon. Second, we apply our measure in a large scale study of long term trends in impact factor biased journal self-citations, based on three decades of citation data for thousands of journals. Our empirical analyses show that impact factor biased journal self-citation practices have become much more prevalent over the past decade. This corresponds well with the time frame during which the impact factor has gained widespread attention among researchers and journal editors [19]. It therefore seems likely that the increase in impact factor biased journal self-citation practices relates to the increasing importance of impact factors and reflects increasingly pervasive journal self-citation malpractices.

## Results

Consider a measure of Impact Factor Biased Self-citation Practices (from here on: IFBSCP). This measure (see Materials and Methods section for a formal derivation and interpretation) compares the share of journal self-citations in year *y* to papers published in impact factor years (i.e., year *y* − 1 and *y* − 2), with the share of journal self-citations to papers published in the five preceding years. The IFBSCP of a journal equals 1 if the share of journal self-citations to papers published in impact factor years equals the share of journal self-citations to papers published in preceding years. An IFBSCP that is (much) above 1 signals a disproportional share of journal self-citations to papers published in impact factor years. Although there may be perfectly legitimate reasons for a high IFBSCP (see Discussion section), it is a possible indication of malpractices such as manipulated editorials, coercive citation practices and authors’ strategic response to such (anticipated) practices. Table 1 (see S1 File and S2 File for the original data sources) reports the share of journals indexed in the Web of Science database which, in a given year, have an IFBSCP higher than a given threshold. It also presents the mean IFBSCP across journals for a given year.

**Table 1:** Trends in IFBSCP since 1987 (all scientific fields combined).

Year	# journals	% of journals with IFBSCP > 1	% of journals with IFBSCP > 1.5	% of journals with IFBSCP > 2	% of journals with IFBSCP > 3	Mean IFBSCP
1987	1520	85.5%	34.4%	12.6%	2.3%	1.47
1988	1572	86.1%	35.3%	10.6%	1.4%	1.45
1989	1580	86.8%	35.0%	11.6%	1.5%	1.45
1990	1618	86.5%	35.2%	13.0%	2.2%	1.48
1991	1664	84.0%	35.5%	11.2%	1.8%	1.44
1992	1694	84.4%	32.4%	10.7%	1.7%	1.42
1993	1742	83.5%	33.5%	11.0%	2.0%	1.42
1994	1775	84.3%	33.5%	10.8%	1.6%	1.43
1995	1851	82.0%	32.6%	10.9%	2.2%	1.44
1996	1995	84.3%	34.2%	11.1%	1.7%	1.44
1997	2040	84.4%	34.8%	11.3%	1.7%	1.44
1998	2087	84.0%	33.3%	11.5%	1.9%	1.44
1999	2182	80.3%	30.7%	10.4%	1.5%	1.39
2000	2216	81.0%	31.0%	10.3%	1.8%	1.40
2001	2340	80.8%	30.0%	10.3%	2.2%	1.39
2002	2462	79.0%	28.6%	9.8%	2.3%	1.38
2003	2543	80.7%	30.0%	9.8%	2.2%	1.40
2004	2686	81.3%	29.3%	10.8%	2.5%	1.41
2005	2818	81.1%	32.6%	12.6%	2.9%	1.44
2006	2956	82.0%	33.6%	12.9%	3.0%	1.46
2007	3124	84.4%	36.0%	14.2%	3.4%	1.51
2008	3276	84.9%	38.2%	15.2%	3.3%	1.52
2009	3454	86.5%	40.1%	16.0%	3.5%	1.55
2010	3590	85.8%	40.3%	16.7%	4.1%	1.57
2011	3779	88.2%	42.4%	16.9%	4.5%	1.63
2012	3979	87.7%	45.3%	18.4%	5.2%	1.66
2013	4145	88.5%	43.5%	18.0%	4.8%	1.65
2014	4460	88.9%	44.7%	18.7%	5.0%	1.64
2015	4767	88.2%	44.6%	18.9%	5.6%	1.68

**Fig 1:**
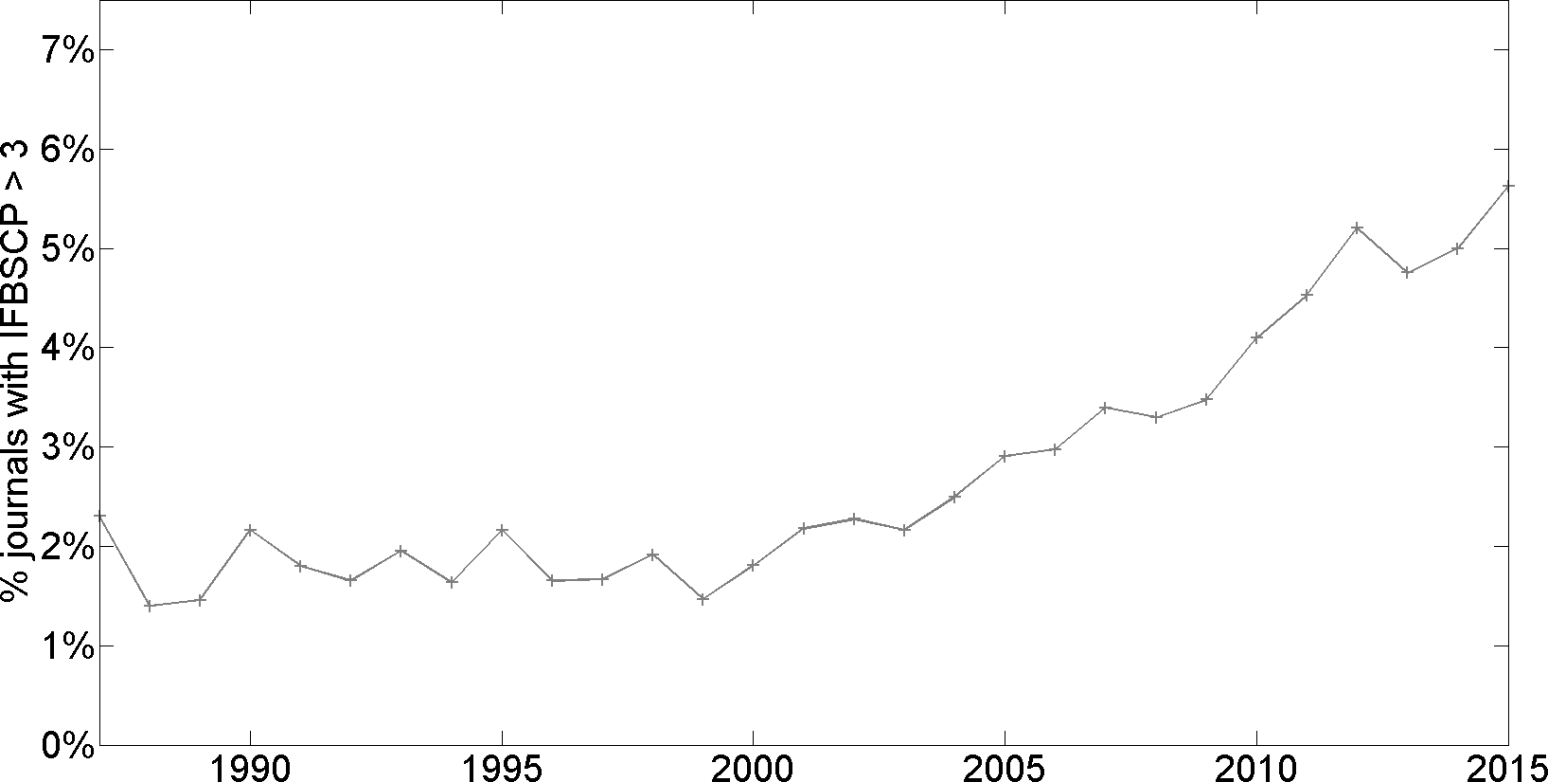
Percentage of journals with IFBSCP > 3 (all fields of science combined).

**Fig 2:**
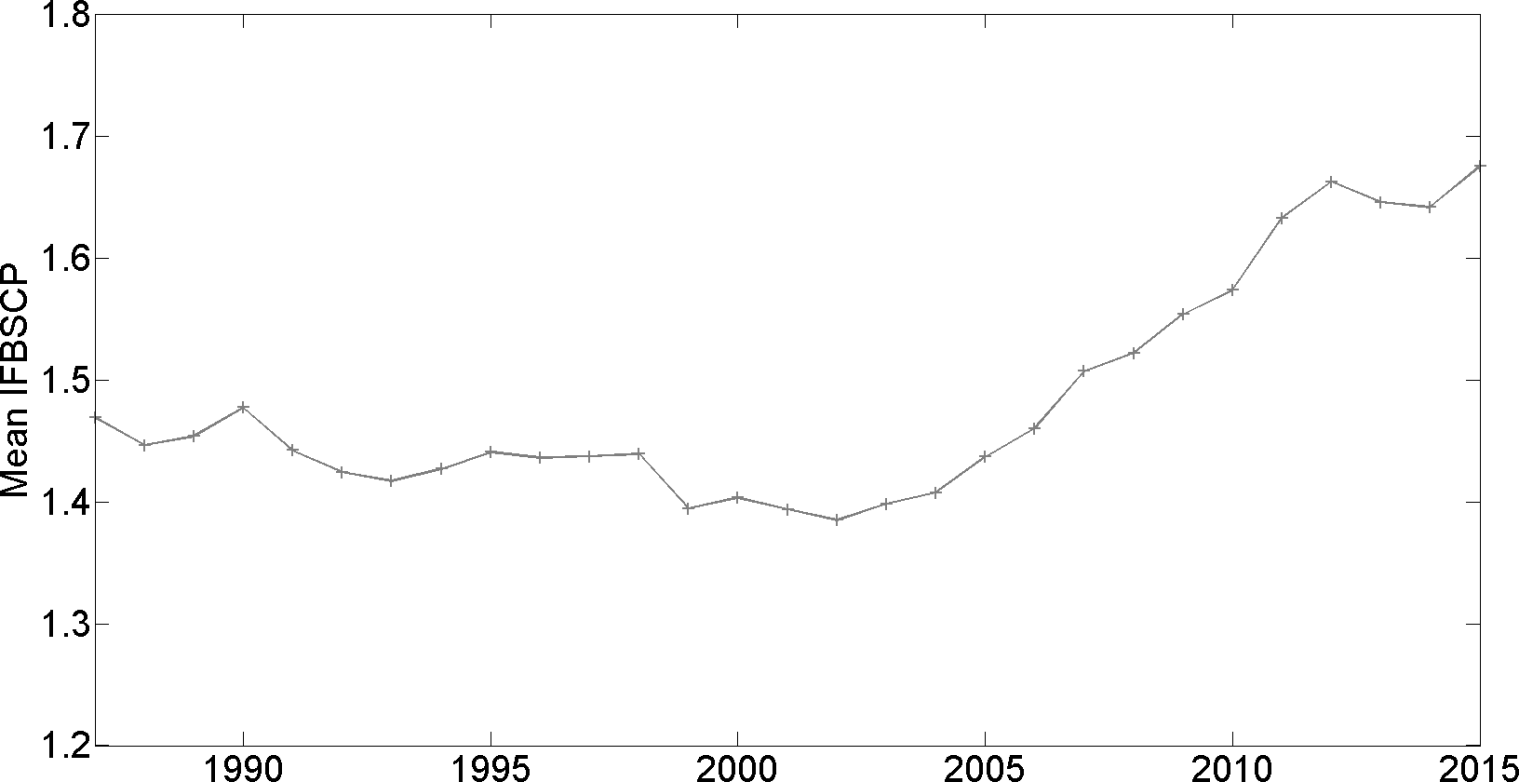
Mean IFBSCP (all fields of science combined).

A first observation is that a large majority of journals have an IFBSCP which is higher than 1; see third column from the left. As we discuss in the Discussion section, this can be explained by various legitimate mechanisms that may trigger an overrepresentation of journal self-citations to recently published papers. Nonetheless, it is directly seen that while the share of journals with an IFBSCP higher than given thresholds was relatively stable from 1987 to around 2004, it has markedly increased since then. For example, while between 1987 and 2004, around 33% of journals had an IFBSCP higher than 1.5, this share has rapidly increased since and is now 45%, implying a rise of more than 35%. This recent rise in the share of journals with an IFBSCP that surpasses a given threshold becomes even more pronounced for higher thresholds: for the threshold of 2, the share is stable at somewhat more than 10% between 1987 and 2004, while growing to almost 19% in 2015. For a threshold of 3, the share was stable at around 2% between 1987 and the early 2000s, and has since almost tripled to 5.6% in 2015. The mean IFBSCP, while being quite stable (and if anything, moderately declining) between 1987 and 2004, has since rapidly increased. Fig 1 provides a visualization of the trend concerning the share of journals with an IFBSCP that surpasses 3; Fig 2 does the same for the trend concerning mean IFBSCP.

**Fig 3:**
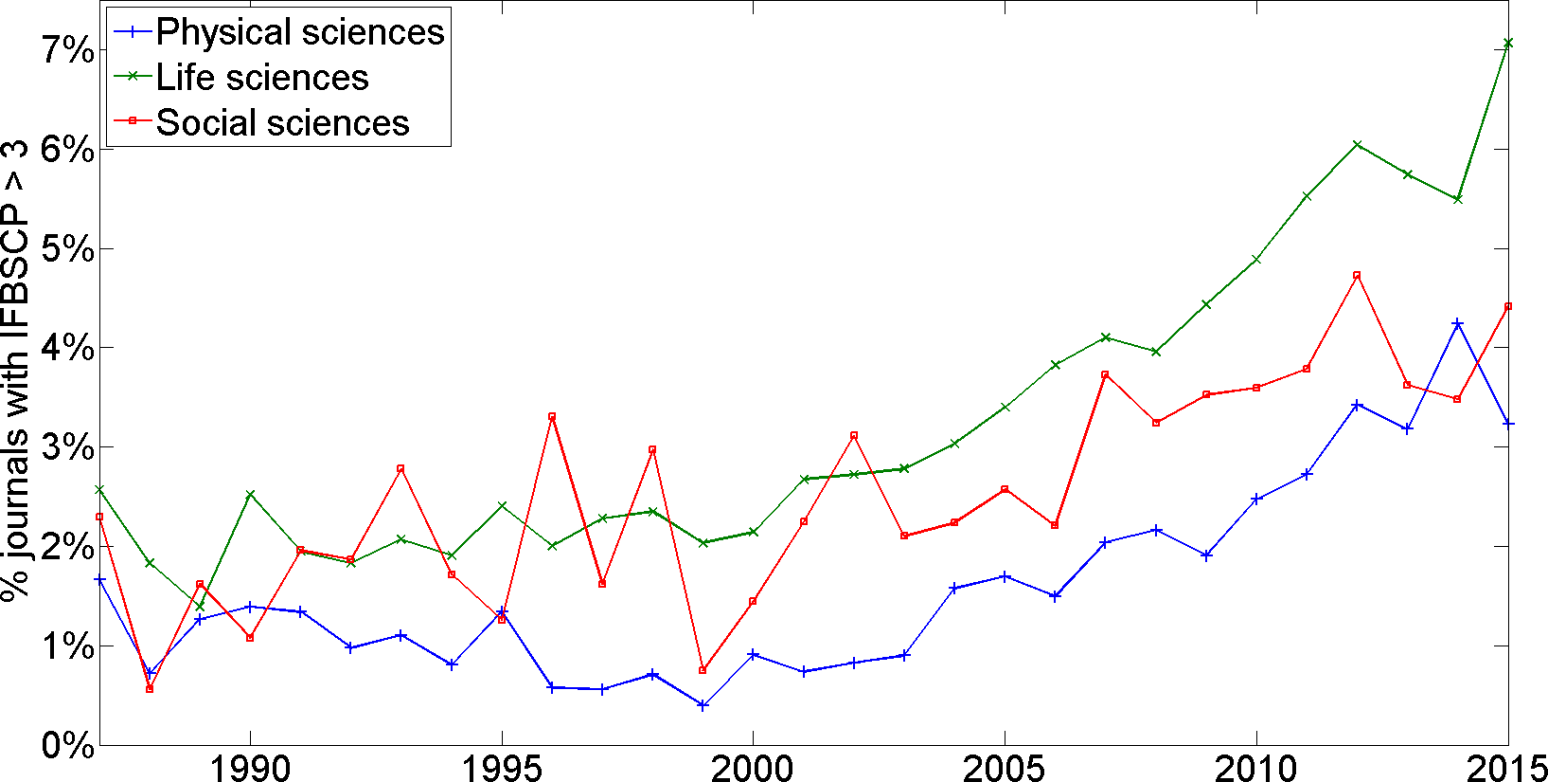
Percentage of journals with IFBSCP > 3 (different fields of science).

**Fig 4:**
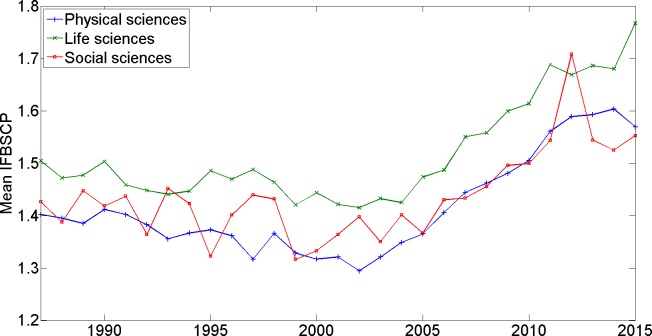
Mean IFBSCP (different fields of science).

Our data allow us to explore if there are any eye-catching differences in (trends in) IFBSCP between the Physical, Life, and Social Sciences. More specifically, Fig 3 presents the time trend in the percentage of journals in a given domain, whose IFBSCP surpasses 3 in a given year. Fig 4 presents the time trend in the mean IFBSCP in a given domain.

Inspection of Figs 3 and 4 warrants the following observations: first, all three fields exhibit the above described trends–more specifically, the pronounced increase in mean IFBSCP and in the percentage of journals with a high IFBSCP over the past decade. Second, compared to the Social and Physical Sciences domains, the Life Sciences domain exhibits a higher mean IFBSCP and a higher share of journals with a high IFBSCP. In particular, in the most recent years, the percentage of journals with an IFBSCP above 3 on average is almost two times higher in the Life Sciences than in the Physical Sciences.

### Relation between IFBSCP and coercive journal self-citations

A survey was held recently, with the aim of exploring how pervasive the malpractice of coercive citation is perceived to be by scholars in selected fields in the Social Sciences, specifically Economics, Sociology, Psychology, and Business ([14]). It was found that about one in every five scholars responding to the survey has experienced coercion him- or herself, and that more than half of the scholars add journal-specific citations before submission. The authors conclude that coercion is “uncomfortably common”. Survey results reported in [14] can be used to study the relation between the IFBSCP measure and a journal’s reputation among scholars in terms of coercive citation practices. More specifically, Table S12 reported in the Supplementary material section in [14], provides a list of “*Journals identified as coercers by survey respondents*. *Number of coercive observations represents the number of times a journal was identified by independent survey respondents as requesting self-citations that (i) give no indication that the manuscript was lacking in attribution*, *(ii) make no suggestion as to specific articles*, *authors*, *or a body of work requiring review*, *and (iii) only guide authors to add citations from the editor’s journal*.” The Table contains 175 journals, from which we selected those 64 journals which met the following conditions: i) the number of coercive observations (as defined directly above) was larger than one, ii) the journal is indexed in the Web of Science database, iii) an IFBSCP value could be computed for the year 2011, going back to 2004 (in other words *Y* = 7 in the IFBSCP equation presented in the Materials and Methods section). We focus on the year 2011 because journals may have altered their practices in response to the publication of [14] in 2012. For each of these 64 journals, the 2011-IFBSCP was computed. Before we can compare the computed IFBSCP with the listed number of coercive observations per journal, the latter measure needs to be normalized to account for differences in journal sizes. More specifically, we divided the number of coercive observations by the number of papers published in a journal in 2011. This gives us a normalized measure of coercive observations per published paper (from here on denoted as COPPP).

The results of our analyses can be summarized as follows (see also Table 2): the mean IFBSCP for the 64 identified journals equals 1.93. To put this into perspective, it should be noted that the mean 2011-IFBSCP for the entire Social Sciences domain (which includes 581 journals with an IFBSCP in 2011) equals 1.54, implying that on average the IFBSCP of the 64 culprits identified in [14] is 25% higher than that of the average Social Science journal. Remember that the survey in [14] focused on Social Science journals. What’s more, 22 out of 64 identified culprits (i.e., more than a third) have an IFBSCP of at least 2.23, which is the 90^th^ percentile threshold for the Social Sciences domain as a whole. Furthermore, when from the list of 64 journals only those 10 with the highest COPPP are considered, their mean IFBSCP equals 2.28; of these 10 journals, 5 have an IFBSCP of at least 2.23, so half of the 10 journals belong to the top 10% journals with the highest IFBSCP in the Social Sciences domain. On the other hand, 2 journals from this ‘top 10’ have an IFBSCP which is lower than the median Social Sciences IFBSCP; these two journals would not have been identified as potential culprits by an IFBSCP-based diagnosis. Finally, unsurprisingly, we find a positive correlation (of 0.19) between the IFBSCP and the COPPP in the population of 64 journals considered.

**Table 2:** Relation between the IFBSCP measure and reported coercive citation practices in [14].

	Mean IFBSCP	Median IFBSCP	% journals with IFBSCP ≥ 2.23 (90^th^ percentile of IFBSCP distribution for Social Sciences journals)
581 Social Science journals	1.54	1.39	10%
64 journals with > 1 coercive observation	1.93	1.81	34%
10 journals with > 1 coercive observation and with highest COPPP	2.28	2.14	50%

In sum, it turns out that the IFBSCP values of journals which were identified in an independent survey as being guilty of coercive citation practices, are considerably higher than those of the average journal in their field. This holds even more so for many, but not all, journals whose coercive citation practices were perceived to be particularly prevalent. These findings suggest that high values of the IFBSCP measure partly relate to coercive citation malpractices. This in turn warrants the conclusion that the IFBSCP measure may be used as a tool of first diagnosis of potential coercive citation malpractices.

Note that the data underlying our analyses are available as supplementary material to this paper (See S1 File and S2 File).

## Discussion

This paper presents a measure, denoted Impact Factor Biased Self-citation Practices (IFBSCP), of the extent to which a journal has a disproportional share of journal self-citations to papers published in years used for computation of the impact factor, compared to papers published in preceding years. Increases in IFBSCP may be due to unethical attempts of journal editors to artificially inflate the impact factor of their journal (potentially in response to pressure from the publisher). For instance, a high IFBSCP may be caused by coercive citation practices ([12], [13], [14], [15]), by editorials with excessive numbers of journal self-citations ([7], [8]), or simply by editors’ acceptance decisions being biased in favor of submissions with many references to papers published recently in their journal. A high IFBSCP may also relate to questionable behavior of authors. Anticipating on the importance of journal self-citations to editors, authors may behave strategically and may include in their paper additional references to papers published recently in the journal to which they plan to submit their work.

However, it cannot be claimed that a high IFBSCP provides proof of unethical behavior of editors or authors. In fact, there are other, perfectly legitimate reasons why a journal may have a relatively high IFBSCP. A recent paper by one of us [18] presents two potential legitimate mechanisms that may trigger an ‘overrepresentation’ of journal self-citations in recent years: First, a reader of a journal may find a paper in its newest issue so interesting, that she decides to do follow up research, e.g. to explore the validity or properties of a recently proposed method, or to test the applicability of a recently reported empirical finding in a different geographical context. She is then of course more likely to submit the resulting paper to this journal, than to another one. This scenario would lead to an increase in the journal’s IFBSCP. Second, an author, after having written a paper, may take a look at her finalized reference list to find out where the most recent papers cited in her reference list have been published. She may consider that the fact that other papers about her paper’s topic were *recently* published in a particular journal is a signal of that journal’s *current* interest in the topic; in line with this consideration, she goes on to submit her work to that journal as opposed to some other journal. This scenario too would lead to an increase in the journal’s IFBSCP. A third potential mechanism which would cause high but legitimate IFBSCP values is as follows: in first instance, the spreading of new ideas is likely to take place in the community where the ideas were originally presented. Over time, the most influential ideas then gradually become noticed and gain traction in other scientific communities. This scenario would imply a relatively large number of journal self-citations in years directly after publication of an idea, compared to more distant (future) years.

Taking into account the above-mentioned legitimate reasons for high IFBSCP values, we stress that a high IFBSCP does not in itself prove wrongdoing by the associated journal. Nonetheless, we do believe that the IFBSCP measure has strong potential as a tool for first diagnosis: it can be used to quickly sift through a large set of candidate journals, and to identify the subset which is relatively likely to include potential culprits. This subset then should be subject to further scrutiny before journal-specific conclusions can be drawn. Our empirical comparison between the IFBSCP measure and the number of reported observations of coercive citation practices in a recent survey [14] provides a justification for this interpretation of the IFBSCP measure: journals identified in [14] as being prone to engage in coercive citation practices generally have a high value on the IFBSCP measure.

We apply the IFBSCP measure to analyze citation patterns for all scientific fields combined since 1987, as well as for the particular domains of Life, Physical, and Social Sciences. We believe that, notwithstanding the caution that should be exercised when interpreting the IFBSCP measure, two over-all conclusions are warranted based on our results: first, between 1987 and the early 2000s, the mean IFBSCP and the share of journals whose IFBSCP surpasses a given threshold has been quite stable; this suggests that there is a stable underlying set of legitimate mechanisms that together result in a mean IFBSCP of around 1.4 at the aggregate level. Second, since about 2004, there has been a pronounced rise in mean IFBSCP and particularly in the share of journals whose IFBSCP surpasses relatively high thresholds (of 1.5, 2, and 3); this suggests that during the last decade, and on top of the legitimate mechanisms discussed earlier, there have increasingly been other influences driving up the IFBSCP, the practices of coercive journal self-citation and strategic responses by authors being obvious candidates.

Although it cannot be ruled out that this steep recent increase in IFBSCP is due to profound changes in the legitimate mechanisms discussed earlier, it should be noted that the importance of the (two year) impact factor has increasingly gained attention among researchers, policy makers, and managers of scientific institutions over the past decade. For example, [19] show that the share of scholarly papers indexed in the Web of Science database mentioning the term “impact factor” in their title has increased steadily since the second half of the 1990s, suggesting a strongly increasing interest in the impact factor since the mid-1990s. Our results show an increasing trend in IFBSCP values starting in the first years of the new millennium. Hence, allowing for a delay of a few years, the increasing trend in IFBSCP values matches remarkably well with the increasing interest in the impact factor. That paper [19] also shows that journals in the Life Sciences publish many more editorials about the impact factor than journals in the Physical and Social Sciences, suggesting that interest in the impact factor is strongest in the Life Sciences. Again, this matches very well with our results, since we find that journals in the Life Sciences are more likely to have high IFBSCP values than journals in the Physical and Social Sciences.

Together, it seems fair to consider the strong rise in mean IFBSCP and in the share of journals with a (very) high IFBSCP, as circumstantial evidence for a trend of increasingly pervasive journal self-citation malpractices, with all due unwanted consequences such as inflated perceived importance of journals and biased journal rankings. We recommend that future studies into such malpractices use the IFBSCP measure to zoom in on a small subset of journals whose IFBSCP is suspiciously high, before embarking on the collection of much needed case study evidence for or against the actual malpractice at the level of particular journals.

## Materials and Methods

### The IFBSCP measure

Assuming that journal self-citation malpractices are aimed at inflating a journal’s impact factor, one would expect that a journal which actively engages in such a malpractice, has a share of journal self-citations to papers published in that journal in recent years that is relatively large compared with the share of journal self-citations to papers published in that journal in earlier years. This is the underlying rationale for the IFBSCP measure, which is formally denoted for a given journal as follows:

$${IFBSCP\;}^{y}=\frac{\left(S_{y-1}^{y}+S_{y-2}^{y}\right)/\left(C_{y-1}^{y}+C_{y-2}^{y}\right)}{\left(S_{y-3}^{y}+\ldots +S_{y-Y}^{y}\right)/\left(C_{y-3}^{y}+\ldots +C_{y-Y}^{y}\right)},$$

where *y* represents the year for which the IFBSCP is computed; $S_{y-1}^{y}$ denotes the number of self-citations from papers published in the journal in year *y* to papers published in the same journal in year *y* – 1; $S_{y-2}^{y}$ denotes the number of self-citations from papers published in the journal in year *y* to papers published in the same journal in year *y* − 2; note that years *y* − 1 and *y* − 2 constitute the two years that are used for computing the journal’s impact factor. $C_{y-1}^{y}$ denotes the *total* number of citations from papers published in any journal in year *y* to papers published in the focal journal in year *y* – 1; $C_{y-2}^{y}$, $S_{y-3}^{y}$, $C_{y-3}^{y}$, etc. are defined likewise, and *Y* stands for reach of the analysis (i.e., *y* − *Y* represents the earliest year included in the analysis).

Three comments need to be made on the above definition of the IFBSCP measure. First, the idea of analyzing the time trend in the ratio of self-citations to total citations is also used in recent studies on journal self-citations and journal citation cartels ([10], [15], [18]). Second, our measure differs from the procedure outlined in [18] in terms of how the denominator is defined; [18] uses all years preceding *y* in the analyses, which generates overlap between the years represented in the numerator and denominator. Third, the IFBSCP measure as defined above assumes that journal self-citation malpractices typically refer to papers from the past two years, which are the years that determine the traditional two year impact factor. Of course, following the same ideas as above, a variant of the IFBSCP measure for the five year impact factor can be easily derived.

### Data

We extract citation data from the Web of Science database for all fields in the Sciences and the Social Sciences. We do not consider fields in the Arts and Humanities. Journals in the Arts and Humanities do not have an impact factor, and presumably editors of these journals therefore have less incentive to engage in coercive self-citation practices. We compute the IFBSCP for every journal and for every year in the period 1987–2015. We set *Y* = 7, so in effect we compare the share of journal self-citations to papers published in ‘impact factor-years’ with the share of journal self-citations to papers published in the five years preceding the ‘impact factor-years’ (see below for a stability analysis, using different values for *Y*). To focus our analyses on journals for which sufficiently reliable statistics can be computed, we exclude those journals that–in a given year–cite their own papers in the past seven years fewer than 50 times. This results in the exclusion of 88,842 out of 164,757 cases, where each case represents a combination of a journal and a year). The total number of journals considered increases from 1520 in 1987 to 4767 in 2015; the total number of cases in the period 1987–2015 being 75,915. For the analyses reported in Fig 3 and Fig 4, we make comparisons across different fields of science: this is based on 366 journals on average for the Social Sciences, 938 for the Physical Sciences, and 1532 for the Life Sciences. Note that we perform no tests of statistical significance of differences across domains and between years, since we are effectively observing the entire population of interest as opposed to a sample thereof. Note also that IFBSCP statistics for all journals included in our analyses are available from the authors upon request. Interested readers can use these statistics for follow-up analyses at the level of individual journals.

### Stability analysis

The decision to set *Y* at 7 (implying a comparison between self-citations for the two impact factor years and the five preceeding years) is somewhat arbitrary. We therefore report the results of a series of stability analyses which we performed to investigate the impact on IFBSCP of setting *Y* at different values. Specifically, we consider the year 2015 (i.e., *y* in the IFBSCP formula equals 2015), and select all journals that continuously published during the previous decade, i.e., in each of the years 2005–2015. This results in 7223 journals. For each of these journals, we compute IFBSCPs for *Y* ∈ {4,5,6,7,8,9,10}. We report three different stability metrics: first, we report the Pearson correlation between the IFBSCP with *Y* set at 7 and each of the alternative specifications. These correlations range from 0.71 (*Y* = 4) to .95 (*Y* = 8); note that it was expected that the highest correlations would be found for values of *Y* close to 7, since the underlying citation data have large overlap. To study stability in rank ordering (i.e., whether the position of a journal in a rank ordering in terms of IFBSCP remains stable), we compute Kendall’s tau correlations as well. These correlations range from 0.69 (*Y* = 4) to 0.90 (*Y* = 8). Finally, we compute–for each journal–the ratio of the IFBSCP for *Y* = 7 and the IFBSCP for *Y* = 4 and *Y* = 10; i.e., we compare *Y* = 7 with the two most extreme values of *Y* in our data. The ratio (IFBSCP for *Y* = 7) / (IFBSCP for *Y* = 4) has a mean (across 7223 journals) of 1.16; the 10-th percentile IFBSCP equals 0.84, and the 90-th percentile IFBSCP equals 1.51. The ratio (IFBSCP for *Y* = 7) / (IFBSCP for *Y* = 10) has a mean (across 7223 journals) of 0.94; the 10-th percentile IFBSCP equals 0.78, and the 90-th percentile IFBSCP equals 1.07. In combination, these analyses indicate that the IFBSCP is stable across different values of *Y*.

## Supporting Information

S1 File Citation data. Contains citation data from which Table 1 is computed.
(XLSX)

S2 File IFBSCP Results. Contains the IFBSCPs reported in Table 1 and Figs 1–4.
(XLSX)
